# Charged Residues Distribution Modulates Selectivity of the Open State of Human Isoforms of the Voltage Dependent Anion-Selective Channel

**DOI:** 10.1371/journal.pone.0103879

**Published:** 2014-08-01

**Authors:** Giuseppe Federico Amodeo, Mariano Andrea Scorciapino, Angela Messina, Vito De Pinto, Matteo Ceccarelli

**Affiliations:** 1 Department of Chemical and Geological Science, University of Cagliari, Cagliari, Italy; 2 Department of Physics, University of Cagliari, Cagliari, Italy; 3 Istituto Officina dei Materiali, Consiglio Nazionale delle Ricerche, Cagliari, Italy; 4 Department of Biological, Geological and Environmental Sciences, Section of Molecular Biology, University of Catania, Catania, Italy; 5 National Institute for Biomembranes and Biosystems, Catania, Italy; University of Saskatchewan, Canada

## Abstract

Voltage Dependent Anion-selective Channels (VDACs) are pore-forming proteins located in the outer mitochondrial membrane. They are responsible for the access of ions and energetic metabolites into the inner membrane transport systems. Three VDAC isoforms exist in mammalian, but their specific role is unknown. In this work we have performed extensive (overall ∼5 µs) Molecular Dynamics (MD) simulations of the human VDAC isoforms to detect structural and conformational variations among them, possibly related to specific functional roles of these proteins. Secondary structure analysis of the N-terminal domain shows a high similarity among the three human isoforms of VDAC but with a different plasticity. In particular, the N-terminal domain of the hVDAC1 is characterized by a higher plasticity, with a ∼20% occurrence for the ‘unstructured’ conformation throughout the folded segment, while hVDAC2, containing a peculiar extension of 11 amino acids at the N-terminal end, presents an additional 3_10_-helical folded portion comprising residues 10′ to 3, adhering to the barrel wall. The N-terminal sequences of hVDAC isoforms are predicted to have a low flexibility, with possible consequences in the dynamics of the human VDACs. Clear differences were found between hVDAC1 and hVDAC3 against hVDAC2: a significantly modified dynamics with possible important consequence on the voltage-gating mechanism. Charge distribution inside and at the mouth of the pore is responsible for a different preferential localization of ions with opposite charge and provide a valuable rationale for hVDAC1 and hVDAC3 having a Cl^−/^K^+^ selectivity ratio of 1.8, whereas hVDAC2 of 1.4. Our conclusion is that hVDAC isoforms, despite sharing a similar scaffold, have modified working features and a biological work is now requested to give evidence to the described dissimilarities.

## Introduction

Voltage Dependent Anion-selective Channels (VDACs) are a small family of conserved proteins mainly located in the outer mitochondrial membrane, whose permeability they guarantee [1]–[3]. They conduct ions, metabolites and small molecules, among which the energetic nucleotides ATP, ADP and NADH, with limitations due to the physical available size of the channel’s conduit [4]. Three different VDAC isoforms have been characterized in higher eukaryotes, encoded by three separate genes [3], [5]. In most cells, VDAC1 is the most abundant isoform, being ten and hundred times more prevalent than VDAC2 and VDAC3, respectively [6]. It is thus not surprising that VDAC1 has been the most extensively characterized isoform. VDAC1 exhibits a single-channel conductance of ∼3.5–4.0 nS in 1 M KCl at an applied voltage between −20 mV and +10 mV [1], [2], [7]. Raising the applied voltage results in the channel switching to the so-called “closed state”, with a lower average conductance and a channel selectivity reversed to cations [4]. In addition to the pore-forming function, VDAC1 is involved in various interactions and cross-talk with other cellular proteins like hexokinase [8], tubulin [9], the Ca^2+^ gating into mitochondria [10] and the Bcl-2 family members [11] that can impact on the activity of the pore itself and vice versa, testimony to the involvement of VDAC to crucial cell fates [12] like in pathways leading to apoptosis [13]–[15], cancer [16], [17] and degeneration [18].

After a long quest [19], the structure of VDAC1 has been recently solved by x-ray crystallography and nuclear magnetic resonance (NMR). It is a large transmembrane channel (outer diameter ∼4.5 nm; inner diameter 2.0–2.5 nm; height ∼4 nm) formed by 19 amphipathic β-strands [20]–[22]. Such an open barrel is made by the regular antiparallel organization of the β-strands, but the parallel pairing of the strands 1 and 19 that completes the channel. Whereas in bacterial porins an even number of antiparallel β-strands is generally observed [23], the structure of VDAC is absolutely peculiar [24]. It is not known whether this exception to the rule of an even number of strands in protein β-barrels might have any influence on the stability and/or functionality of VDAC, and it has to be reported that several criticisms have been raised against this structure and whether it is the actual native conformation [25]. On the other hand, a series of evidences have been reported in the literature to support the 19-strands structure against the previous models [26], [27]. In addition, molecular dynamics simulations have shown that the x-ray/NMR solved structure is compatible with the experimental values of both conductance and ions selectivity of the open state [28], [29].

**Figure 1:**
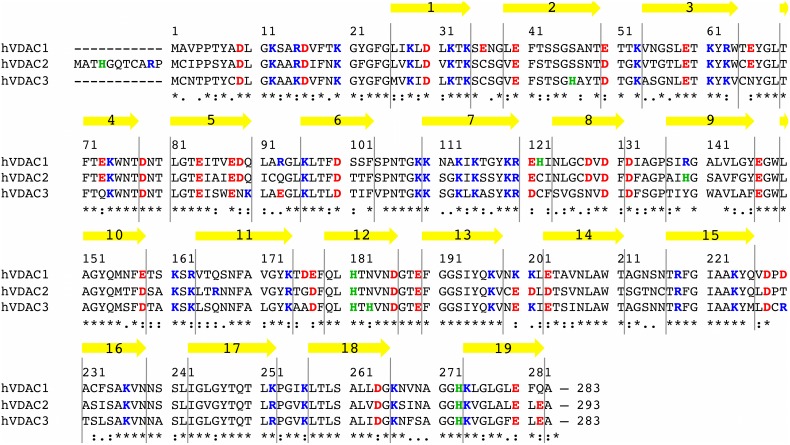
Multiple sequence alignment of the three isoforms of hVDAC. Residues are numbered according to hVDAC1 and hVDAC3 sequence (283 AA), thus, the first 11 N-terminal residues of hVDAC2 (293 AA) are not numbered in the figure and are referred to as 1′-11′ throughout the paper. Acidic residues are colored in red, basic ones in blue. Histidines have been distinguished from the latter and colored (green). The yellow arrows show the 19 β-strands forming the barrel. (Sequence alignment was obtained through ClustalW2 at http://www.ebi.ac.uk/Tools/msa/clustalw2/).

The amino acid sequence of VDAC is highly conserved. The three human isoforms (hVDAC), in particular, are 68% to 75% pairwise identical, with 80–90% overall similarity. Figure 1 shows the sequence alignment of the three human isoforms of VDAC. The high sequence homology among the hVDAC isoforms (figure 1) has been interpreted as a similarity among the respective 3D structures. Thus, the 3D structure of the VDAC2 and VDAC3 isoforms have been predicted on the basis of secondary structure prediction servers [5]. It was only during revision of the present work that a crystallographic structure of VDAC2 was published [30] (zebra fish VDAC2; PDB code 4BUM at 2.8 Å resolution) confirming the very high degree of structural similarity with the VDAC1. The most striking difference between the three human isoforms is certainly the longer N-terminal fragment of hVDAC2, which has 11 residues more than the other two isoforms (figure 1). The N-terminal fragment of VDAC, comprising the first 25 amino acid residues (36 in the case of hVDAC2), is located inside the channel in the 3D structures, partially closing the wide pore [20]–[22]. However, differences exist among the reported structures and they mainly refer to the N-terminus. This is due to a sum of factors like, for example, the temperature used in the NMR or in the crystallographic collection of experimental data. In human [20] and mouse [22] VDAC1, the N-terminal fragment crosses the lumen and is folded as an α-helix from the residue 6 to 20, broken at the conserved residue G11. This folded portion is actually amphipathic, with the more hydrophobic residues directed towards the barrel wall, while the hydrophilic ones point the lumen center. The same sequence shows a mostly unfolded conformation in the structure reported by Hiller [21], but it has to be pointed out that this structure was obtained with NMR, at room temperature.

In this work, microseconds all atom MD simulations were performed in order to compare the intrinsic dynamic behavior of the three human isoforms of VDAC. The channel electrostatics was deeply analyzed and it was found how mutation of key residues affects ions preferential localization inside the lumen, thus influencing selectivity. The hVDAC2 was found to be significantly different from the other two human isoforms, in terms of both electrostatics and dynamics of the pore.

## Results and Discussion

### General aspects

Simulations were carried out both in the absence of KCl (apart from the few counterions needed to neutralize the system total charge) and in the presence of KCl 0.5 M. No transmembrane potential was applied in all the cases. KCl was the electrolyte of choice due to the almost equal diffusion coefficient of chloride and potassium ions in dilute solutions [31]. For small ions, the translocation through a relatively large protein channel such as the VDAC is expected to depend on their mobility in water, the electrostatics of the lumen and the protein dynamics. Thus, by using ions with comparable mobility, it is possible to focus on the properties of the protein. No significant differences were observed in the structural features and dynamics of each human VDAC isoform when KCl 0.5 M was added in our simulations. Thus, the results obtained for the three proteins in the presence of KCl will be reported, compared and discussed hereinafter.

**Figure 2:**
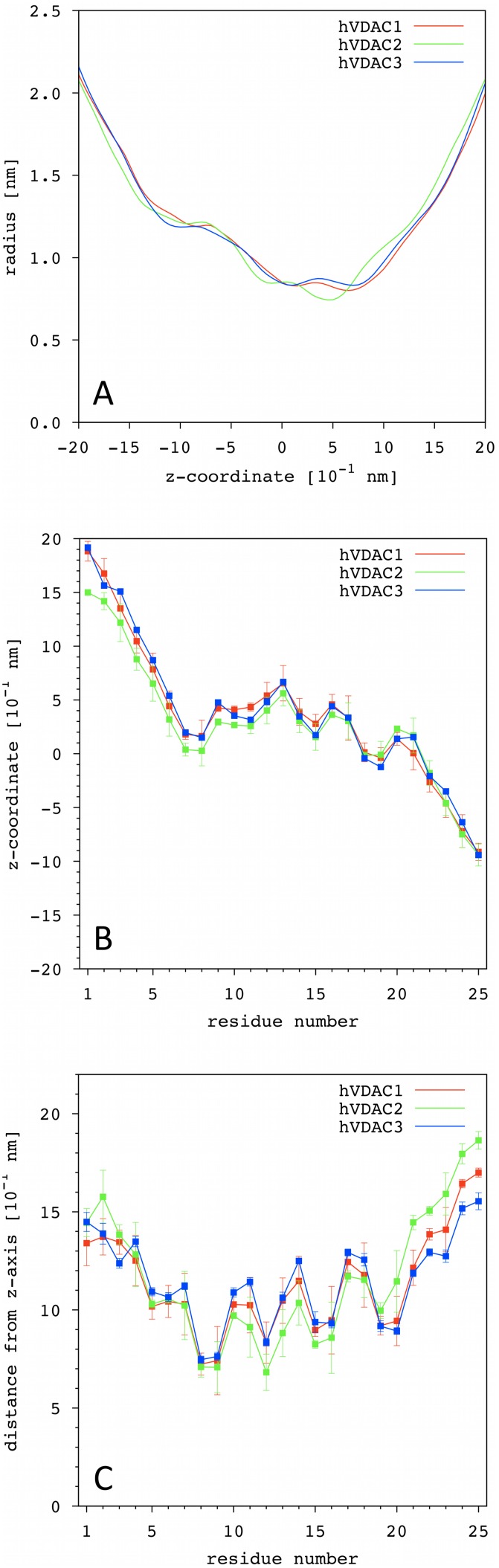
Channel radius and position of the N-terminal domain inside VDAC lumen. (**A**) Lumen radius calculated from solvent accessible area as a function of the z-coordinate for the three isoforms of hVDAC. Protein channel is aligned and centered with respect to the z-axis, with both the N- and C-terminus located on the positive-z side of the lipid bilayer. (**B**) The z-coordinate and (**C**) the distance from the z-axis of the Cαs is shown. Error bars were calculated as the standard deviation over 5 independent MD replicas. The residues 1′-11′ of the hVDAC2 are not shown for the sake of clarity; they are exposed to the solvent outside the lumen.

Figure 2A shows the lumen radius as a function of the z-coordinate. In our simulations the protein is oriented along the z-coordinate with both the N- and C-terminus located on the positive-z side of the lipid bilayer. Recently, using intact cells, it has been demonstrated that the C-terminus faces the mitochondrial inter-membrane space [32]. All isoforms are not symmetric, with the positive-z half of the lumen characterized by a steeper decrease of the radius than the negative half. The absolute minimum is not located at z = 0, indeed. Both hVDAC1 and hVDAC3 have the minimum at z ∼6.7 10^−1 ^nm and a radius ∼0.82 nm, while it is located at z = 4.7 10^−1 ^nm in hVDAC2 with a significantly lower radius of 0.74 nm. A second local minimum is found to be close to the lumen center in all VDACs with a comparable value of ∼0.83 nm, whose precise location, again, is different for hVDAC2 (z = −1.0 10^−1 ^nm) with respect to the other two isoforms (z ∼1.14 10^−1 ^nm).

### The N-terminal domain in the three isoforms

The N-terminal domain of VDAC is considered a strategic asset for protein stability and functionality. It has been shown to be crucial for channel gating [25], [26], [28], [29], [33]–[37] as well as for interaction with apoptosis related proteins [3], [15], [38], [39]. The N-terminal domain has been observed to be characterized by a rather low flexibility, and the key role in stabilization of VDAC barrel in the open state has been put forward [28], [34], [37], [40].

In the mVDAC1 reported by Ujwal [22], and similarly in the hVDAC1 structure proposed by Bayrhuber [20], the N-terminal adheres to the barrel wall on the side of strands 8–16, located approximately at the midpoint of the hydrophobic portion of the membrane. It faces the very few hydrophobic residues directed towards the channel interior [24], [34], [40], [41]. In addition, H-bonds contribute to facilitate its interaction with the interior wall of the pore [22], [28], [41]. In the mVDAC1 structure in particular [22], two hydrogen bonds are observed between the carbonyl oxygens of residues A2 and P4 and the backbone nitrogen of H122 and the Nδ of N124, which are located on the wall of the pore. Although the helical hydrogen-bonding pattern is broken at L10 and G11, separating the helix into two segments, these two segments are capable of maintaining a rigid structure because R15 forms bidentate hydrogen bonds with the carbonyl oxygens of A8 and L10. In addition, the helical portion on the N-terminal side has two hydrogen bonds to β-strands 12 and 16, stabilizing its interaction with the wall of the pore.

A close inspection of the amino acid sequence of the three human isoforms (figure 1) reveals intriguing differences. The hVDAC2 has the first 11 residues not aligned with the sequence of hVDAC1 and hVDAC3. The residue numbers from the latter will be used throughout the paper, while the first 11 residues of hVDAC2 will be referred to as 1′-11′. Furthermore, different mutations are present in the N-terminal sequence. In particular, whereas hVDAC1 has no cysteines, both hVDAC2 and hVDAC3 are characterized by the presence of two cysteines in this protein region. It is not known whether they form disulfide bridges with other cysteines located in the barrel wall under physiological conditions. This would clearly affect the N-terminal mobility and, in turn, the flexibility of the barrel, and might represent a striking difference in the dynamics of the human isoforms of VDAC. In the present investigation, all cysteines were simulated in the reduced form, since the molecular analysis of refolded VDAC2 suggested, indeed, that the cysteines do not form disulfide bridges [42].

Another interesting difference among the sequence of the three hVDACs is the distribution and the number of acidic and basic residues. Neglecting histidines, that were not charged in our simulations, all the three isoforms have a net positive charge: hVDAC1 has 32 basic residues and 29 acidic ones (net charge +3), hVDAC2 contains 30 basic and 29 acidic (net charge +1), hVDAC3 has 31 basic and 25 acidic (net charge +6). Mutations are present either in the loops and in the barrel forming β-strands, but the N-terminal sequence shows a remarkable conservation of the distribution of charged residues with prevalence of the positive ones (figure 1).

Figures 2B–C show the position of the α-carbons comprising the N-terminal fragment of the three hVDAC isoforms. Either the z-coordinate (2B) and the distance from the z-axis (2C) is shown. Residues 1′–11′ of the hVDAC2 are not shown for the sake of clarity; they were exposed to the solvent outside the lumen. This is because such additional residues of hVDAC2, as said, are not aligned neither with the amino acid sequence of the other two isoforms (Figure 1), nor with the mVDAC reference structure used as template for the homology modeling (see the Methods). Thus, the software used to generate the starting configuration of the three hVDACs did not apply any geometrical restraint on these residues. This resulted in their simple addition to the protein structure model according to a random coiled backbone conformation. Recently [30], the crystallographic structure of VDAC2 has been solved for zebra fish (PDB code 4BUM) at high resolution (2.8 Å), showing a very high degree of similarity with both the human and mouse VDAC1 3D structure. The root mean square deviation (rmsd) between zebra fish VDAC2 and the mVDAC reference structure used in this work is only 0.98 Å. The zebra fish VDAC2 amino acid sequence has 84% identity and 98% similarity with the human VDAC2, with the main differences located in the external loops. In agreement with the solved 3D structures, the N-terminal fragment crosses the lumen with a helical folding from the residue 6 to 20 in all the isoforms, and is horizontally located close to the lumen center. In the hVDAC2, the first half of the folded fragment is characterized by slightly lower values for both the z-coordinate and the distance from the z-axis, providing an explanation for the different profile of the lumen radius vs z-coordinate obtained (figure 2A). However, differences are only slight and might be due to some inaccuracies in the modeling of the very first residues of hVDAC2, by virtue of presence of the additional 11 amino acids commented above.

The hydrophaticity profile of the N-terminal sequence (figure S1) is conserved in the three human isoforms, with the folded part characterized by a similar pattern of alternate hydrophobic and hydrophilic residues. The comparison between this profile and the distance of the α-carbons from the z-axis (figure 2C) clearly shows that the N-terminal domain directs the hydrophilic residues towards the lumen center and protects the hydrophobic ones from the water solvent by facing the channel wall, in agreement with previous experimental observations [43].

In all the three isoforms the most hydrophilic residues are the number 12, 15, 16, 19 and 20, whose mutation has been shown to affect channel selectivity and voltage-gating [35], [44]. Whereas the most hydrophobic ones are the number 10, 17 and 18. The only striking difference among the three VDAC isoforms is given by residue 3 (V, I and N in hVDAC1, 2 and 3, respectively), which is markedly hydrophobic in both hVDAC1 and hVDAC2, while it is mutated into a strongly hydrophilic residue in hVDAC3.

shows the channel wall lined by the N-term helical fragment with the residues side chain color coded on the basis of their hydrophobicity score. Our comparative study shows that all the three human isoforms share the hydrophobic contacts between the N-terminal domain and the few inward directed hydrophobic residues of the channel wall.

Secondary structure analysis of the N-terminal domain (figure S3) shows a high similarity among the three human isoforms of VDAC with a 3_10_-helix comprising the residues 6–12 and an α-helix from residue 13 to 20. However a different plasticity was observed in our simulations. The hVDAC2 and hVDAC3 are comparable with a somewhat rigid α-helical fragment and a low (∼10%) occurrence for the ‘unstructured’ conformation in the 3_10_-helical portion. On the other hand, the N-terminal fragment of the hVDAC1 is characterized by a higher plasticity, with a ∼20% occurrence for the ‘unstructured’ conformation throughout the folded segment. As far as the hVDAC2 is concerned, it is interesting to note the additional 3_10_-helical folded portion comprising residues 10′ to 3, which, being amphipathic, adheres to the barrel wall. This results in a slight tightening of the main folded part of the N-terminal fragment, as it is shown in more details hereinafter.

Position and orientation of the N-terminal fragment in the VDAC lumen do not only depend on its amphipathicity and hydrophobic contacts with the barrel wall, but also on specific hydrogen bonds [22], [28]. Table 1 reports the H-bonds formed by the residues of the N-terminal fragment having an occurrence >20% in our simulations. All the three human isoforms of VDAC are characterized by the presence of the most stable H-bonds between the very first residues of the N-terminal fragment and the residues located at the barrel border, namely, in the strands (or in the β-turns) 8–11. Despite this appears to be a conserved features of all the three VDAC isoforms, the occurrence and the number of these H-bonds resulted to be significantly lower in the case of hVDAC1 (Table 1). These results complement the secondary structure analysis. A weaker anchoring of the N-terminal fragment and, in turn, a higher plasticity are observed for the hVDAC1 when compared to the other isoforms.

**Table 1:** aHydrogen bonds formed by residues of the N-terminal fragment.

hVDAC1									hVDAC2									hVDAC3								
res.1		at.1	at.2	res.2		occ.^b^ [%]			res.1		at.1	at.2	res.2		occ.^b^ [%]			res.1		at.1	at.2	res.2		occ.^b^ [%]		
									R	10′	Hη	Oε	E	147	66.9	±	8.7									
																		M	1	H	Oδ	D	121	63.3	±	1.8
A	2	O	H	H	122	41.7	±	4.5	C	2	O	H	C	122	66.0	±	4.3									
									C	2	Sγ	H	E	147	54.7	±	6.5									
									I	3	O	Hη	R	174	44.3	±	5.9	N	3	H	Oδ	D	121	42.0	±	7.3
																		N	3	Hδ	Oδ	D	121	28.7	±	6.1
P	4	O	Hδ	N	124	41.9	±	10.2	P	4	O	Hδ	N	124	35.7	±	4.4	T	4	Hγ	O	R	120	74.3	±	10.8
																		P	5	O	Hγ	S	124	28.2	±	8.6
T	6	Hγ	Oδ	D	9	50.9	±	24.0	S	6	Hγ	Oδ	D	9	46.9	±	5.6	T	6	Hγ	Oδ	D	9	89.0	±	2.0
																		T	6	H	Oδ	D	9	34.1	±	4.8
A	8	O	Hη	R	15	33.6	±	7.7	A	8	O	Hη	R	15	28.4	±	2.5									
D	9	Oδ	Hη	R	15	36.1	±	15.8																		
L	10	O	Hη	R	15	29.7	±	5.6	L	10	O	Hη	R	15	32.5	±	6.2	L	10	O	Hζ	K	15	26.1	±	1.3
									K	12	Hζ	Oδ	D	16	35.8	±	4.7									
R	15	O	Hγ	T	19	37.1	±	11.9	R	15	O	Hδ	N	19	24.9	±	3.8									
D	16	Oδ	Hζ	K	224	43.4	±	20.0										D	16	Oδ	Hζ	K	224	34.2	±	7.3
D	16	Oδ	Hζ	K	20	26.9	±	2.4	D	16	Oδ	Hζ	K	20	31.8	±	4.9									
V	17	O	Hη	Y	22	69.8	±	4.7										V	17	O	Hη	Y	22	65.8	±	5.0
F	18	O	Hζ	K	236	63.7	±	2.8	F	18	O	Hζ	K	236	65.6	±	2.2	F	18	O	Hζ	K	236	67.1	±	11.9
G	25	H	O	L	275	68.8	±	6.7	G	25	H	O	V	275	50.8	±	8.0	G	25	H	O	V	275	53.3	±	3.0
									G	25	O	H	T	49	28.2	±	4.8	G	25	O	H	T	49	25.4	±	2.9

a H-bonds involving two residues of the N-terminal fragment are reported in *italics*, in order to be distinguished from those involving one residue of the N-term and one of the barrel. N-term backbone-backbone H-bonds are not reported for the sake of brevity, since they simply reflect the secondary structure analysis (figure S5).

b Only H-bonds with an occurrence >20% are reported. Error is given as the standard deviation over 5 independent MD replicas.

The conserved H-bond between F18 and K236 is also remarkable. It is located right at the end of the folded portion of the N-terminal fragment and contributes to stabilize its position and orientation inside the lumen. Almost all the models proposed in the literature for VDAC voltage-dependent gating involve a more or less extended displacement of the N-terminal fragment, which, bearing a net positive charge, would sense the transmembrane voltage. Whether it ends up outside the lumen and completely exposed to the solvent [15], or bound to the membrane surface [45], or it simply detaches form the barrel wall but stays inside the pore [22], [26], [28], [40], the interactions between the N-terminal fragment and the barrel play a fundamental role in stabilizing the open over the closed state of the channel, as it has been also shown by β-strand deletion experiments [41].

### Channel breathing motions

**Figure 3:**
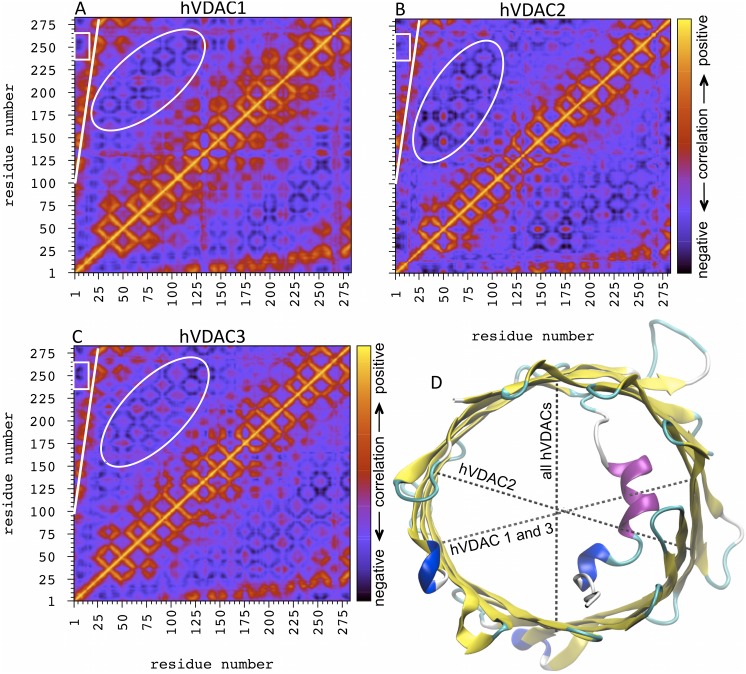
Correlated fluctuations of hVDACs β-barrel. Correlation map of α-carbons fluctuations of (**A**) hVDAC1, (**B**) hVDAC2 and (**C**) hVDAC3. The map is color coded as reported in the box, moving from positive (yellow) to negative (dark blue) correlation. The positive correlation stripe between the N-terminal fragment and the portion of the barrel wall close to it, is indicated by the white straight line. The white oval circumscribes a series of anti-correlation spots between opposite sides of the barrel. The small white rectangle indicates the position of another interesting anti-correlation spot between the very beginning of the N-terminal fragment and the opposite side of the pore. (**D**) The corresponding axes (dashed lines) of maximal anti-correlation are shown for the three hVDAC isoforms using the structure of hVDAC1 as a template.

A comprehensive picture of the existing interconnection between the fluctuations of the N-terminal fragment and the channel walls is achieved through the α-carbons correlation map. Figure 3 shows that, from a general point of view, the correlation map is very similar for the three hVDAC isoforms. A stripe of positive correlation spots is present between the N-terminal fragment and the residues of the β-strands 8–19 as expected (indicated by the white straight line in figures 3A–C), due to the above mentioned interactions between the N-terminal domain and this side of the β-barrel.

On the other hand, a clear anti-correlation spot is observed between the very beginning of the N-terminal fragment and the region around the residue 250 (indicated by the white rectangle in figures 3A–C), which corresponds to the opposite side of the pore with respect to the position where the N-terminus is ‘anchored’ to the β-barrel (see above). This position is exactly located on the vertical defined by residue 25, i.e. where the N-terminal domain is bonded to the first β-strand. In other words, a sort of anti-correlation axis can be defined for all the hVDAC isoforms as shown in figure 3D, which, running longitudinally to the N-terminal direction through the lumen, connects two regions of the channel wall that are close to the beginning and the end of the N-terminal domain, respectively.

Interestingly, almost all β-barrel residues showing a positive correlation with the N-terminal fragment, i.e. those located in the β-strands 8–19 (see above), have a remarkable negative correlation with the residues located on the other side of the channel, as shown by the series of anti-correlation spots indicated by the white oval in figures 3A–C. Thus, taking the central residues of the N-terminal fragment as reference and the corresponding β-barrel residues with the highest positive correlation, we looked for the maximum anti-correlation spots. This way, a second anti-correlation axis was identified for the three hVDAC isoforms as shown in figure 3D, which, running transversally to the N-terminal direction through the lumen, connects opposite regions of the channel wall. As shown in figure 3D, the direction of this transverse axis is different for hVDAC2 with respect to hVDAC1 and hVDAC3.

The overall shape of the β-barrel cross-section can be described as elliptic with one axis being longitudinal and the other transversal to the N-terminal fragment. For each hVDAC isoform we conveniently chose a series of residues (their α-carbon), at three different heights with respect to z-axis of the pore, in order to evaluate the length of these two axes as a function of simulation time. The Pearson cross-correlation coefficients between such distances are reported in table S1. All isoforms are characterized by positive correlation between the axes with the same directionality, bolstering the analysis of the α-carbons correlation map and showing that the barrel walls fluctuate almost uniformly throughout the z-coordinate. However, our analysis revealed that hVDAC2 has a significantly different dynamics. Fluctuations of the longitudinal and transversal axis of the pore are significantly anti-correlated in hVDAC1 and hVDAC3, whereas hVDAC2 shows almost no correlation between fluctuations of the two axes.

Recently, such anti-correlated elliptical fluctuations of the β-barrel has been proposed in the literature on the basis of an extensive computational and experimental investigation of hVDAC1 [28]. The N-terminal domain was shown to have a relatively low mobility and to play a fundamental role in the modulation of β-barrel rigidity. Deletion of the N-terminal fragment led to a marked ellipticity of the channel (and higher fluctuations), mostly achieved through the shortening of the distance between the β-strands 1 and 9, exactly corresponding to the ‘longitudinal axis’ defined in the present work. Correspondingly, a slight elongation of the transverse axis was observed. The authors [28] concluded that elliptic deformation of the barrel is an essential component of voltage-gating and that changes in anion selectivity strictly depend on the specific shape of the channel (related to the charges distribution inside the lumen).

**Figure 4:**
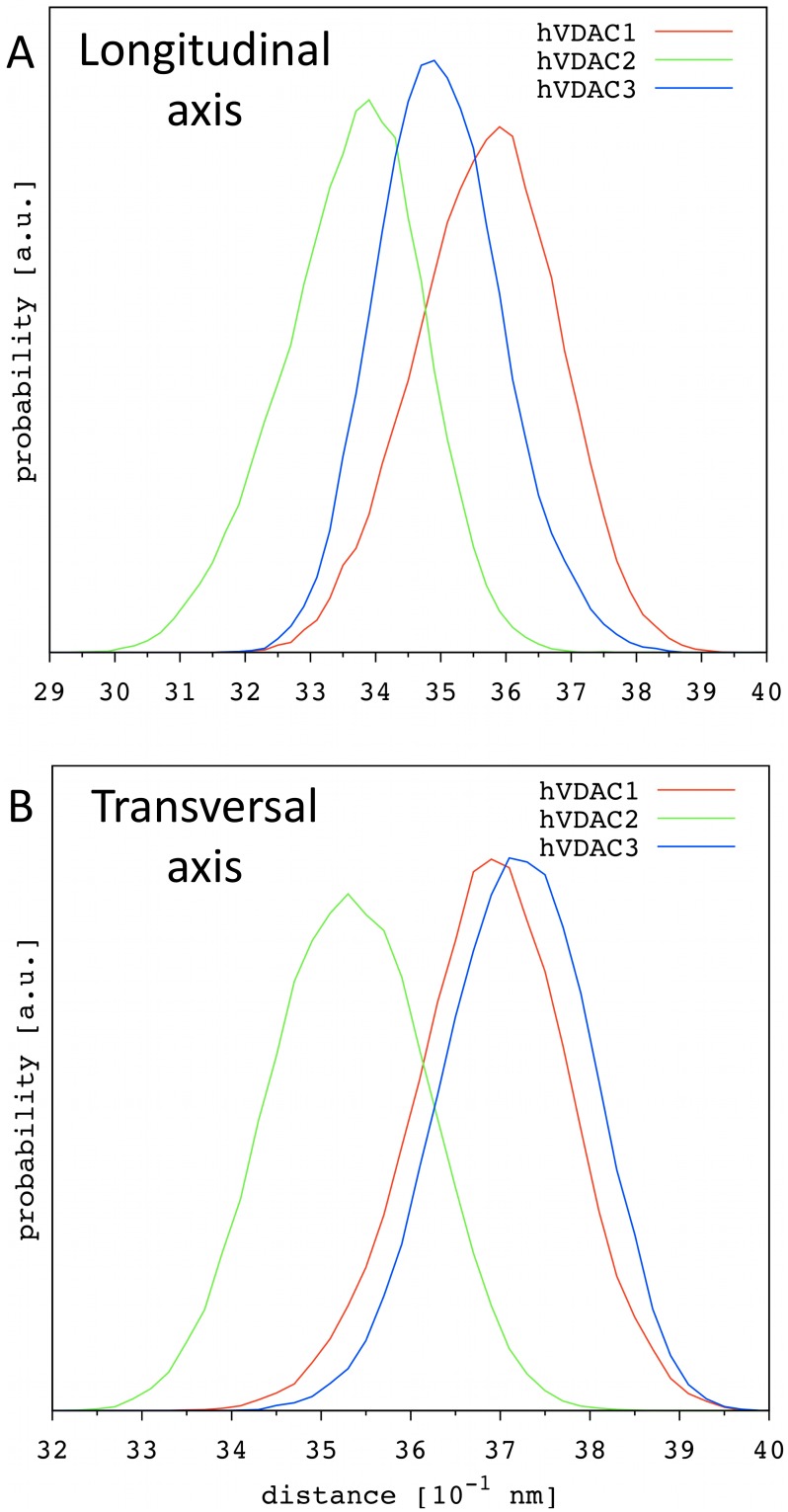
Probability distribution of the two axes used to describe the channel elliptic shape. Correlation analyses led to define two major axes of elliptic breathing motions of the β-barrel. The one is longitudinal the other is transversal to the N-terminal fragment direction through the pore. Probability distribution is shown for the (**A**) longitudinal and (**B**) transversal distance calculated at z ≈0 from 5 independent MD replicas for the three human isoforms.

Our structural characterization shows that such elliptic movements of the barrel are an intrinsic feature of all hVDAC isoforms but hVDAC2. Even in the presence of the N-terminal domain they are evident and represent the spontaneous breathing motions of this protein channel at equilibrium. Figure 4 shows the probability distribution of the length of both the longitudinal and transversal axis calculated for the three hVDACs. The longitudinal axes follow the order hVDAC1>hVDAC3>hVDAC2 (3.59, 3.49 and 3.39 nm, respectively; figure 4A). These results perfectly match the relative number and occurrence of the H-bonds formed between the very first N-terminal residues and the barrel reported above. The hVDAC2 showed the most stable interactions and its N-terminal fragment is characterized by the presence of an additional 3_10_ helical portion that adheres to the barrel wall. Its tighter N-terminal fragment forces the barrel longitudinal axis to shorter values than observed for the other two isoforms.

The transversal axes are in the order hVDAC3 ≈hVDAC1>hVDAC2 (3.72, 3.69 and 3.53 nm, respectively; figure 4B). In agreement with the correlation analyses, the transversal axis is found to be longer than the corresponding longitudinal one, showing that the overall shape of the channel is often slightly elliptical. Ellipticity values are in agreement with those reported in the literature for the wild-type hVDAC1 [28]. Despite hVDAC2 has the shortest longitudinal axis among the three isoforms, it is not characterized by the longest transversal axis. This is not surprising, since no correlation has been found between the two distances in this case, but it is interesting to note that hVDAC2 results to be the more compact isoform with both the longitudinal and transversal axis being shorter, on average, than observed for the other two human isoforms. Definitely, a strong interplay between the N-terminal fragment flexibility and β-barrel motions emerged, in agreement with other observations reported in the literature [28], [29]. VDAC voltage-gating mechanism has been proposed to depend upon more or less extended movements of a highly charge portion of the protein [25], [26], [28], [29], [33]–[37]. Any motion of a charged mobile segment should occur along the direction of the electrical field applied [46]. Despite the present work was performed in the absence of any trans-membrane voltage, we can hypothesize that the switch to the closed state of VDAC is hindered if the N-terminus remains inside the channel. Thus, a movement of the N-terminal helix out of the lumen should be the very first step of the voltage-gating mechanism.

### Ions Passive Translocation

Table 2 summarizes the results obtained for the three hVDAC isoforms in the absence of transmembrane voltage. As expected from the literature [38], [46], [47], all the hVDACs resulted to be slightly selective for anions. The hVDAC1 and hVDAC3 are characterized by Cl^−/^K^+^ selectivity ratio of 1.8, whereas hVDAC2 is less selective with a ratio of 1.4. The value of 1.8 found for hVDAC1 is in very good agreement with the values reported in the literature, obtained from both experimental and computational investigations on hVDAC1 and/or mVDAC1 [1], [33], [48]. The lower average value obtained for hVDAC2 is in agreement with the experimental observation of two distinct populations for this particular isoform, one with similar conductance and selectivity to VDAC1, the other with a lower conductance and anion selectivity [47], [49].

**Table 2:** aIons translocation and selectivity in the absence of transmembrane voltage.

		hVDAC1		hVDAC2		hVDAC3	
Cl^−^ inside^b^		7.7±0.2		6.8±0.2		7.5±0.3	
K^+^ inside^b^		4.4±0.3		4.9±0.3		4.2±0.2	
Anion selectivity^c^		1.7±0.2		1.4±0.1		1.8±0.0	
Cl^−^ to K^+^ correlation^d^		+0.8±0.1		+0.7±0.1		+0.8±0.0	
**TRANSLOCATION EVENTS** e **(number of events; average time [ns])**							
Cl^−^	↓	51±6	2.2±0.1	42±8	2.0±0.2	43±5	2.2±0.2
Cl^−^	↑	44±2	2.2±0.3	40±6	2.1±0.2	49±8	2.3±0.2
K^+^	↓	16±4	2.3±0.4	19±3	2.4±0.5	20±3	2.5±0.3
K^+^	↑	18±5	2.2±0.6	16±2	3.1±1.3	20±4	2.1±0.3
Permeation ratio^f^		2.8±0.3		2.4±0.2		2.3±0.3	

a Error is given as the standard deviation over 5 independent MD replicas.

b Average number of ions per frame found inside the lumen (−18≤ z ≤ +18).

c The time averaged ratio between Cl^−^ and K^+^ found inside the lumen.

d Cross-correlation coefficient between the number of Cl^−^ and K^+^ found inside the lumen as a function of simulation time.

e The arrow indicates the direction (z) of the translocation.

f The overall ratio between Cl^−^ and K^+^ translocation events.

From our simulations, this difference appears to be mostly due to a different overall affinity of hVDAC2 for chloride ions. On average, ∼7.6 Cl^−^ have been found inside hVDAC1 and hVDAC3, while the hVDAC2 mean value was lower by ∼1 (Table 2). On the other hand, the difference in the average number of K^+^ found inside the lumen was smaller and within the limit of the statistical error of our simulations. A high positive correlation was found between the number of oppositely charged ions inside the lumen as function of simulation time, suggesting that channel affinity for Cl^−^ and for K^+^ are not mutually independent. Indeed, ion-pairing has been observed in previous computational investigations [50], and it has been put forward that K^+^ needs Cl^−^ to travel through the pore.

Looking at the actual translocation events (table 2), there is no significant difference comparing the results obtained for the +z and –z direction in all the three hVDAC isoforms, thus, the Cl^−/^K^+^ permeability ratio has been calculated taking the events altogether into account. A slightly higher value is obtained for hVDAC1 than for the other two isoforms, even if the difference is within the limit of the statistical error of our simulations. The values found for the permeability ratio are in agreement with those reported in the literature [51]. A closer inspection of the overall number of Cl^−^ and K^+^ translocations reveals that, similarly to the number of ions found inside the lumen, the number of potassium events is comparable for all the isoforms, while hVDAC2 shows a lower number of chloride events than the other two hVDACs.

It is interesting to note that, for all the isoforms, no significant differences have been found in the average translocation time of Cl^−^ and K^+^ in both directions, suggesting a negligible difference in the overall translocation kinetics of the two ions and further supporting the major role played by channel affinity in anion-selectivity.

**Figure 5:**
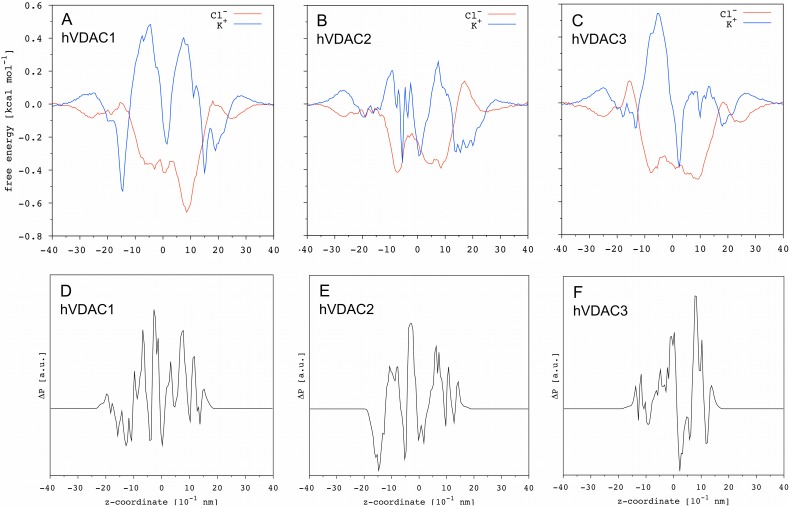
Ions free energy profile and charged residues distribution. Chloride and potassium profiles are shown for (**A**) hVDAC1, (**B**) hVDAC2 and **(C)** hVDAC3 as function of the z-coordinate. The statistical error estimated over 5 independent MD replicas is ∼0.1 kcal mol^−1^. In the panels (**D–F**) the difference ΔP_z_ between the distribution of positively and negatively charged residues facing the protein lumen is reported as function of the z-coordinate.

Figures 5A–C show the free energy profiles obtained for Cl^−^ and K^+^ in the three hVDAC isoforms. From a general point of view, the shape of the free energy profiles is comparable for the three proteins investigated as well as to the profiles reported in the literature [48]. Chloride is characterized by a mostly negative free energy with two wells at z ∼−10 and +10, respectively, separated by a modest barrier. Potassium profile is somewhat complementary, being mostly positive and characterized by a sharp well at z ∼0 and two broad energy barriers around z ∼-10 and +10, respectively. Chloride integrated ΔG (−18< z < +18) follows the order hVDAC1 ≈hVDAC3<hVDAC2 (−21.4, −19.5 and −14.7 kcal mol^−1 ^nm, respectively), which correlates with the opposite order found for the time-averaged number of Cl^−^ inside the pore (table 2). Potassium integrated ΔG follows the order hVDAC1 ≈hVDAC3>hVDAC2 (+4.8, +5.2 and −2.7 kcal mol^−1 ^nm, respectively), which correlates with the opposite order found for the time-averaged number of K^+^ inside the pore (table 2). For all the three isoforms, the difference between the height of the maximum energy barrier, taking either chloride and potassium and both directions into account, is <<1 kcal mol^−1^. Indeed, no significant difference is observed between the average translocation time (Table 2).

However, it is very informative to analyze and compare the specific differences between the free energy profiles obtained for the three hVDAC isoforms. On both sides of the channel (+30> z > +20; −20> z > −30), chloride experiences a free energy decrease upon approaching the protein, due to the overall net positive charge of the latter, while potassium shows a small barrier. On the contrary, at the lumen entrance (+20> z > +15; −15> z > −20), a small energy barrier is observed for chloride and a well for potassium. While these two barriers for chloride (and the two complementary potassium wells) have a comparable height in hVDAC1, the profile at the two entrances is not symmetric for hVDAC2 and hVDAC3.

The hVDAC1 has two negatively charged residues more than the positive ones at both entrances, explaining the comparable small free energy barrier for chloride. However, the number of charged residues at the entrances is not the same, the one at positive-z having 14 charged residues (8 negative and 6 positive), while the other has only 8 charged residues (5 negative and 3 positive). The energy barrier seems to be not determined by the number of charged residues per se, but mostly by the counterbalance between positively and negatively charged amino acid residues.

The hVDAC2 is characterized by a stronger unbalancing at the positive-z entrance, with 7 negatively and only 4 positively charged residues and, indeed, the barrier for chloride is higher on that side.

The case of hVDAC3 is even more informative. Despite the equal number of positively and negatively charged residues at the two entrances, it has a higher chloride energy barrier at the negative-z entrance (figure 5C). Charged residues are not uniformly distributed in this case. One negative residue does not have a positive counterpart in its proximity, resulting in local charge unbalance. Thus, while the net charge as function of the z-coordinate is certainly important in the determination of ions free energy profile, the charges distribution with respect to the x- and y-coordinate appears to be not negligible to achieve a comprehensive picture.

In an attempt to explain the differences observed among the three hVDACs, we first computed the distribution of positively and negatively charged residues along ‘z’ and then calculated the parameter ΔP_z_ as the difference between the former and the latter (figure 5D–F). In the case of hVDAC1, ΔP_z_ provides a quite convincing explanation for both chloride and potassium free energy profiles. Around z ∼+10, ΔP_z_ oscillates between ∼0 and positive values, explaining the presence of the deepest chloride well and a high barrier for potassium. Around z ∼−10, ΔP_z_ oscillates as well but shows a higher number of negative regions. Accordingly, the chloride well is less pronounced but, at the same time, ΔP_z_ does not explain why the difference in the height of the two main energy barriers for potassium is negligible. Moving to hVDAC2 and hVDAC3 discrepancies are even more severe. For instance, the barrier separating the two main chloride wells in hVDAC2 corresponds to the highest positive peak of the ΔP_z_ profile, such that one should expect a deep minimum. In the hVDAC3, as another example, the chloride well at z ∼−10 corresponds to a lumen section with ΔP_z_ close to zero. Similarly, ΔP_z_ does not justify the potassium profiles.

### Ions preferential localization inside the lumen

shows the electrostatic potential surface for the three hVDAC isoforms (bottom view of the conformer obtained after the first 200 ns of NVT production run). The highest density of positive potential is observed around the structured segment of the N-terminal fragment. On the other hand, the highest density of negative potential is observed on the opposite side of the channel wall. These features are shared by the three isoforms and are in agreement with results reported in the literature [29], [33]. However, a remarkable difference was found in the central section of the channel on the side of β-strands 6–8, approximately half-way between the highest positive and the highest negative region. In particular, a mostly positive potential was found for hVDAC1 and hVDAC3, whereas a mostly negative potential was observed for hVDAC2.

**Figure 6:**
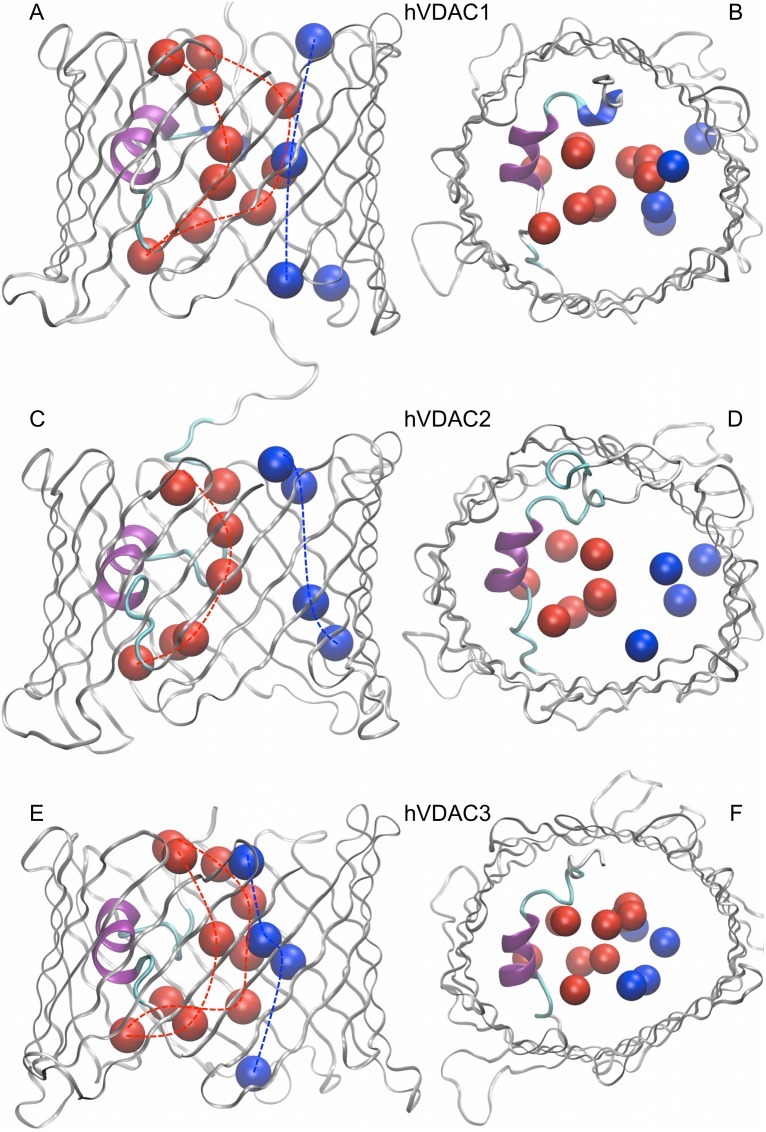
Ions clusters analysis. Clusters (occurrence ≥20%) of chloride (red) and potassium (blue) positions from 5 independent MD replicas are shown for (**A, B**) hVDAC1, (**C, D**) hVDAC2 and (**E, F**) hVDAC3. Both a side (**A, C, E**) and a top view (**B, D, F**) is shown for each isoform. Clusters of each kind were found to be aligned in a sort of path (dotted lines).

A cluster analysis was performed on all the ions coordinates recorded along our 5 independent MD replicas. Figure 6 shows both chloride and potassium clusters (occurrence ≥20%) inside the three hVDAC isoforms. In each case, chloride ions resulted to be preferentially located in the middle of the pore, aligned along a sort of curved path around the N-terminal fragment. On the other hand, the main clusters of potassium ions are localized at the periphery of the lumen, closer to the channel wall and approximately in front of the N-terminal fragment. These results are in perfect agreement with the electrostatic potential surface of the hVDAC isoforms (figure S4): chlorides are preferentially located near the area with the highest density of positive potential, whereas potassium ions are preferentially found next to the channel wall with the highest density of negative potential. Similar observations are reported in the literature for the main isoform [29], [33].

However, both hVDAC1 and hVDAC3 are characterized by a double array of chloride clusters, one of the two being very close to the potassium array, whereas only a single array of chloride clusters was observed in hVDAC2, such that chloride and potassium preferential localization appears to be more clear-cut in this case. This difference is particularly interesting and agrees with all of the above reported results. Indeed, the hVDAC2 is characterized by a lower anion selectivity than the other two isoforms (table 2), due to a significant reduction in chloride affinity. The localization of the ‘missing chloride array’ strongly supports the major role played by the channel electrostatics, since it faces the area of the channel wall where a significant difference in the electrostatic potential has been observed (figure S4), differentiating hVDAC2 (more negative, thus repelling the chloride ions) from the other two isoforms (more positive).

**Figure 7:**
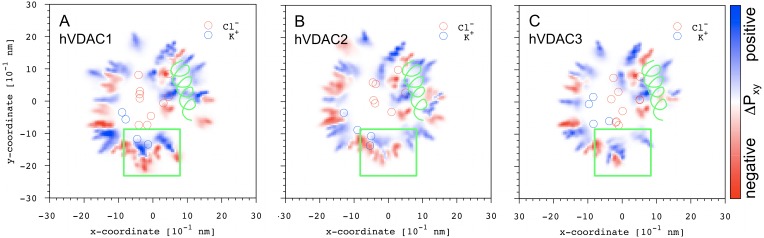
Charged residues distribution on the xy-plane. The difference ΔP_xy_ between the distribution of positively and negatively charged residues on the xy-plane is shown for the three hVDAC isoforms. The green helix represents the N-terminal fragment crossing the lumen, whereas the green rectangle is used to highlight the region with the most striking difference between (**B**) hVDAC2 and (**A, C**) hVDAC1 and hVDAC3.

The parameter ΔP_xy_ was calculated as the difference between the distribution of the positively and the negatively charged residues on the xy-plane. Figure 7 shows the results obtained for the three hVDAC isoforms together with the position of the ions clusters. The green rectangle highlights the area where the most striking difference was observed, in agreement with the electrostatic potential surface (figure S4). In the case of hVDAC2, positive density is significantly reduced when compared to the other two isoforms, one of the two chloride clusters arrays is missing and, in turn, segregation of the preferential localization of chloride and potassium ions is clear.

The residues lining each of the ion clusters were classified into different categories as reported in table 3. Chloride ions clusters are characterized by more positively than negatively charged residues as expected, while potassium ions clusters are lined by an unexpected relatively high number of positively charged residues in all the hVDAC isoforms. This difference is absolutely compatible with the different affinity observed for chloride and potassium ions and provide a valuable additional information to detail VDAC anion-selectivity. Our analysis shows that a number of charged residues line both chloride and potassium clusters. If these ‘promiscuous’ residues are not taken into account, chloride clusters are still lined by more positive than negative amino acids, while potassium clusters are still characterized by more negatively than positively charged residues (table 3). The number of positive ‘promiscuous’ amino acids is higher than the negative ones, contributing to determine the overall selectivity for the anions. The difference between the number of these positive and negative ‘misplaced’ residues in hVDAC2 is significantly smaller than for the other two isoforms and, indeed, hVDAC2 is characterized by only one chloride clusters array, a more clear segregation between the preferential localization of positive and negative ions inside the lumen, and a lower anion selectivity.

**Table 3:** The number of the positively (P) and negatively (N) charged residues lining the ion clusters.

	hVDAC1	hVDAC2	hVDAC3
***CHLORIDE IONS CLUSTERS***			
P average	3.8	4.4	3.0
N average	1.6	1.9	1.5
P total	18	12	15
N total	7	7	9
***POTASSIUM IONS CLUSTERS***			
N average	2.4	2.8	2.6
P average	2.6	3.0	3.6
N total	12	12	10
P total	12	10	13
***SUMMARY***			
P in Cl^−^ clusters only	8	7	4
N in Cl^−^ clusters only	3	4	2
N in K^+^ clusters only	8	9	3
P in K^+^ clusters only	2	5	2
P in both Cl^−^ and K^+^	10	5	11
N in both Cl^−^ and K^+^	4	3	7

Finally, the presence of a relatively higher number of positively charged residues around the potassium clusters, than the negatively charged amino acids found around the chloride clusters, suggests that the cations need anions inside the lumen to counterbalance such positive residues. This is in agreement with [50], where ion-pairing was investigated in detail, and provides a valid explanation for the high positive correlation we found between the time averaged number of Cl^−^ and K^+^ in the lumen (table 2).

## Conclusions

In this work we performed an in-depth analysis of extensive MD simulations of the human VDAC isoforms. The urgency for this work stems from the raising interest in the function of this small family of mitochondrial proteins. The evolution produced three different genes encoding for three different proteins with apparently similar properties. The three VDACs are almost ubiquitously expressed, thus their existence cannot be explained in terms of tissue-specificity. The sequence comparison and the secondary structure predictions suggest a highly conserved 3D structure in the three isoforms. The analysis of the subtle structural and conformational variations among them is thus the best way to understand any functional specificity in these proteins.

We can affirm from our analysis that changes in the amino acid sequence correspond to structural differences with a potential impact on specific functional features. This implies that VDAC isoforms, despite sharing a similar scaffold, have modified working features and a biological work is now requested to give evidence to the found dissimilarities.

It is noticeable that the N-terminal fragment of hVDACs, a very intriguing domain, suspected to be involved in the interaction with other proteins, is characterized by a rather low flexibility in the three isoforms. The presence of cysteines, especially abundant in VDAC2 and VDAC3, raises the suspect that some disulfide bridges might be formed, affecting the N-terminal mobility and, in turn, the flexibility of the β-barrel. This may represent a striking change in the dynamics of the human isoforms of VDAC. On the basis of the overall reducing state of the surrounding fluids, it has been put forward that the cysteines mostly exist in the reduced form [42], thus we have simulated them in such a state. Secondary structure analysis of the N-terminal domain shows a high similarity among the three human isoforms of VDAC but with a different plasticity. In particular, the N-terminal domain of the hVDAC1 is characterized by a higher plasticity, with a ∼20% occurrence for the ‘unstructured’ conformation throughout the folded segment, while hVDAC2, containing a peculiar extension of 11 amino acids at the N-terminal end, presents an additional 3_10_-helical folded portion comprising residues 10′ to 3 that adheres to the barrel wall.

The MD simulations of the whole isoforms revealed that none of them is symmetric, with the positive-z half of the lumen characterized by a steeper decrease of the radius than the negative half. The longitudinal and transversal axes of the pore are not identical and show a clear difference between hVDAC1 and hVDAC3, where they are significantly anti-correlated, with respect to hVDAC2 that shows a significantly different dynamics. This feature has important consequences on the voltage-gating mechanism described as an elliptic deformation of the barrel [28]. Our structural characterization shows that such elliptic movements of the channel wall are an intrinsic feature of all hVDAC isoforms but hVDAC2. However, it has to be stressed here that the present work is based upon model structures, due to the lacking of a X-ray and/or NMR structure for all the three hVDACs at high resolution. The differences we observed are certainly interesting and thought-provoking but still needs to be experimentally confirmed. The road to take is extremely exciting and calls for further investigations. However, during the revision of our work, the 3D structure of zebra fish VDAC2 was solved, confirming the reliability of our homology model.

As far as the charge distribution inside and at the mouth of the pore is concerned, a feature directly affecting the ion flow through the channel, we found that hVDAC1 and hVDAC3 are characterized by Cl^−/^K^+^ selectivity ratio of 1.8, whereas hVDAC2 is less selective with a ratio of 1.4. This was shown to be mainly due to the channel electrostatics. Channel affinity plays a major role in anion-selectivity, as shown by a negligible difference in the average translocation time of Cl^−^ and K^+^ in both directions.

In conclusion this work has the ambition to offer the framework and the structural basis to develop experimental strategies for the final elucidation of the puzzling question about the presence of multiple VDAC isoforms in the cell.

## Methods

The experimental structure of mVDAC1 obtained with X-ray crystallography was used as the starting configuration (PDB code 3EMN at 2.3 Å resolution) [22], having a higher resolution with respect to the 2JK4 available for hVDAC1 [20]. The three hVDAC isoforms were built by homology modeling using the Modeller v9.10 software [52], [53].

The protein was embedded in a pre-equilibrated POPE hydrated bilayer. (i) Lipids were eliminated to create a pore with a radius of 2 nm, (ii) the protein was inserted, (iii) the system was oriented in order to center protein at the origin of the coordinate system and align the channel with respect to the z-axis, (iv) additional lipid molecules at a distance <2 Å from the protein were removed. A suitable number of chloride ions were added in order to neutralize system total charge. The edges of the simulation box were initially 82×82×95 Å, with ∼170 lipids and ∼10000 water molecules (total number of atoms ∼56000). Both the N- and the C- protein termini were located on the positive-z side of the bilayer.

After 1 ps of energy minimization to remove bad contacts, a slow heating from 10 to 300 K was carried out for 1 ns. During this stage, positional restraints were applied on the protein alpha-carbons (all three dimensions) as well as on the lipids phosphorus atoms (along z only). Then, an equilibration stage follows for 10 ns in the NPT ensemble at 1.0 bar and 300 K allowing for waters and lipids rearrangement and box equilibration. Finally, 1.0 µs MD simulations were performed in the NVT ensemble by using the box size reached during the NPT equilibration stage (81.58×81.58×84.25 Å for hVDAC1, 81.86×81.86×83.09 for hVDAC2 and 81.86×81.86×83.03 for hVDAC3). The first 200 ns were considered part of the equilibration stage, while the last 800 ns were used for the analyses.

The NPT equilibration MD simulations were performed with the program NAMD [54], with 1.0 fs time-step, and treating long-range electrostatics with the Soft Particle Mesh Ewald (SPME) method (64 grid points and order 4 with direct cutoff at 1.0 nm and 1.0 Å grid-size). Pressure control was applied using the Nose-Hoover method (extended Lagrangian) with isotropic cell, integrated with the Langevin Dynamics (200 fs and 100 fs of piston period and decay, respectively). The latter was also applied for temperature control with 200 fs thermostat damping time.

Production runs in the NVT ensemble were performed through the ACEMD code [55] compiled for GPUs. The code allowed to rescale hydrogen mass to 4 amu and to increase the time-step up to 4.0 fs (see ref. [56] for instance). The Langevin thermostat was used with 1 ps damping coefficient. SPME was used to treat electrostatics as done during the equilibration stage. Simulations were restarted every 200 ns with new randomly generated velocities. Random numbers generator seed was also changed every restart in order to introduce additional noise and achieve better sampling of the conformational space. This procedure should prevent the system from being trapped in a single potential basin [57].

All MD simulations were performed employing the Amber99SB-ILDN force field [58] for the protein and lipids, and the TIP3P [59] for waters.

### Passive Ions Diffusion

Additional production runs were performed for each hVDAC isoforms in the presence of 0.5 M KCl. The starting coordinates were taken from the frame corresponding to 200 ns of the above mentioned NVT simulations. Using the same parameters reported hereinbefore, we re-equilibrated the system (after KCl addition) in the NPT ensemble for 2 ns and then we moved to the NVT ensemble for 200 ns. Starting from the last configuration, we performed 5 independent 100 ns long MD replicas with different initial velocities.

### Analyses

Channel radius was calculated for each 0.5 Å section normal to the z-axis from the solvent accessible area, using a probe with a radius of 1.4 Å [60]. Residues hydrophobicity scores were obtained with the method of Kyte and Doolittle [61] using a window of 3 residues and a relative weight of the window edges of 30% when compared with the window center. Hydrogen bonds, secondary structure analysis and correlation maps were obtained with the Simulaid package [62]. Protein electrostatic potential surfaces were computed with the Adaptive Poisson-Boltzmann Solver (APBS) tool [63] within the Visual Molecular Dynamics (VMD) software [64]–[67].

Free-energy profiles for chloride and potassium ions were calculated according to the following equation [68]:
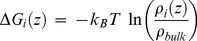
(1)where ΔG(z) is the free-energy as function of the z-coordinate, k_B_ is the Boltzmann constant, ρ_i_(z) the ions density as functions of the z-coordinate and ρ_bulk_ is the averaged ions density outside the pore.

In order to investigate preferential localization of oppositely charged ions in the hVDAC lumen, a cluster analysis was applied to the coordinates of chloride and potassium ions along the MD simulations (−20≤ x,y,z≤20), with a rmsd of 6.0 Å and minimum occurrence of 10%. Basically, the positions recorded along the entire trajectory for all the ions of the same type are used. The number of neighbors within the given rmsd value are counted for each position. The position with the highest number of neighbors is taken together with its neighbors to define the most populated cluster. All the positions assigned to the first cluster are then eliminated from data pool and the procedure is iteratively repeated to look for the other clusters.

## Supporting Information

Figure S1 **Hydrophobicity profile of the N-term fragment.** The hydrophobicity scores were obtained with the method of Kyte and Doolittle [61] and normalized between 0 and 1.
(TIFF)

Figure S2 **Hydrophobic contacts between the N-terminal helical fragment and the channel wall.** Residues’ side chains are colored coded on the basis of the hydrophobicity score calculated with the method of Kyte and Doolittle [61]: the darker the color the more hydrophobic the residue. The position of the more hydrophobic residues comprising the N-terminal fragment are indicated by the yellow arrows, showing the hydrophobic contacts between the N-terminal helix and the channel wall. The hydrophobic contacts between the most hydrophobic residues of the N-terminal fragment and the few inward directed hydrophobic residues of the channel wall are evident, namely, residue 10 interacting with 143 and 150, and residues 17–18 interacting with 205 and 222.
(TIFF)

Figure S3 **Secondary structure of the N-terminal fragment.** Secondary structure is shown for each amino acid residue as the average occurrence of 3_10_-helical, α-helical or unordered conformation over 5 independent MD replicas. The three panels are conveniently placed to reproduce the correct sequence alignment between **(A)** hVDAC1, **(B)** hVDAC2 and **(C)** hVDAC3.
(TIFF)

Figure S4 **Electrostatic potential surface.** The three isoforms are represented from the bottom with positive potential in blue and the negative one in red. The green line represents the position of the N-terminal fragment inside the lumen, whereas the green arrow indicates the area with the most remarkable differences.
(TIFF)

Table S1 **Pearson cross-correlation coefficients for different axes describing the elliptic shape of the channel.**
(DOCX)
