# Supplementary figures and images for: Charged Residues Distribution Modulates Selectivity of the Open State of Human Isoforms of the Voltage Dependent Anion-Selective Channel
Source: PLoS One. 2014 Aug 1;9(8):e103879. doi: 10.1371/journal.pone.0103879 (PMC4146382; doi:10.1371/journal.pone.0103879)

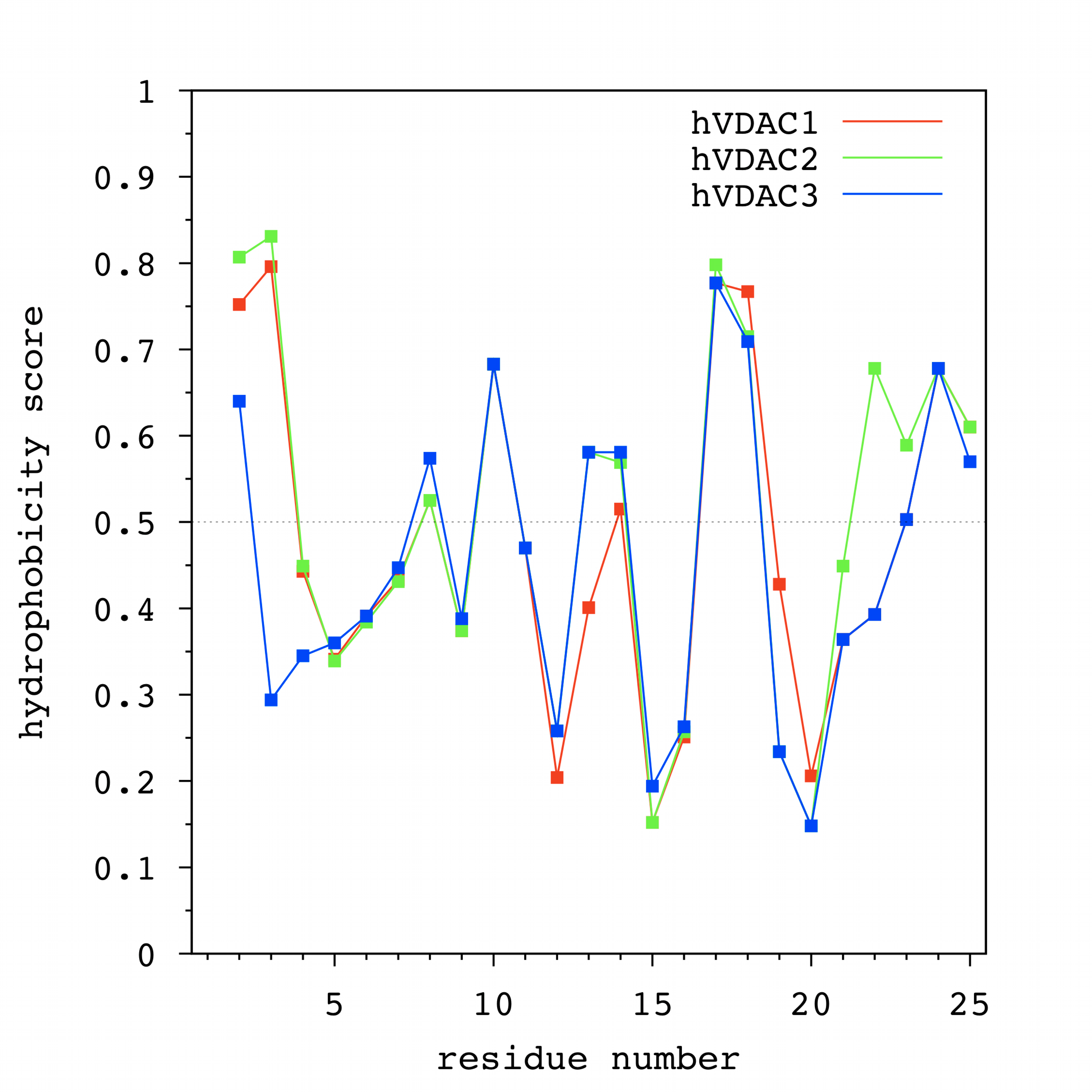

Supplement: Figure S1 — Hydrophobicity profile of the N-term fragment. The hydrophobicity scores were obtained with the method of Kyte and Doolittle [61] and normalized between 0 and 1. (TIFF) [file pone.0103879.s001.tiff]

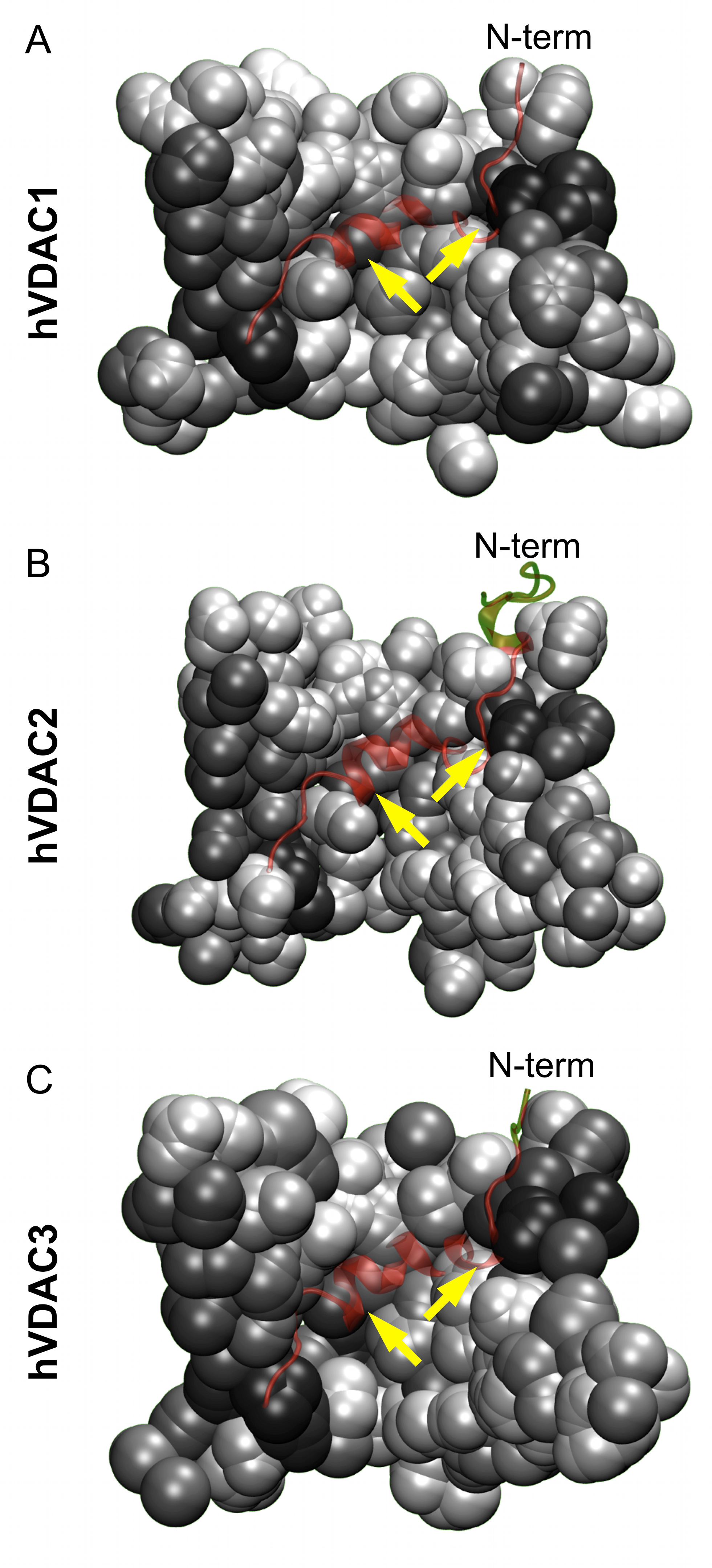

Supplement: Figure S2 — Hydrophobic contacts between the N-terminal helical fragment and the channel wall. Residues’ side chains are colored coded on the basis of the hydrophobicity score calculated with the method of Kyte and Doolittle [61]: the darker the color the more hydrophobic the residue. The position of the more hydrophobic residues comprising the N-terminal fragment are indicated by the yellow arrows, showing the hydrophobic contacts between the N-terminal helix and the channel wall. The hydrophobic contacts between the most hydrophobic residues of the N-terminal fragment and the few inward directed hydrophobic residues of the channel wall are evident, namely, residue 10 interacting with 143 and 150, and residues 17–18 interacting with 205 and 222. (TIFF) [file pone.0103879.s002.tiff]

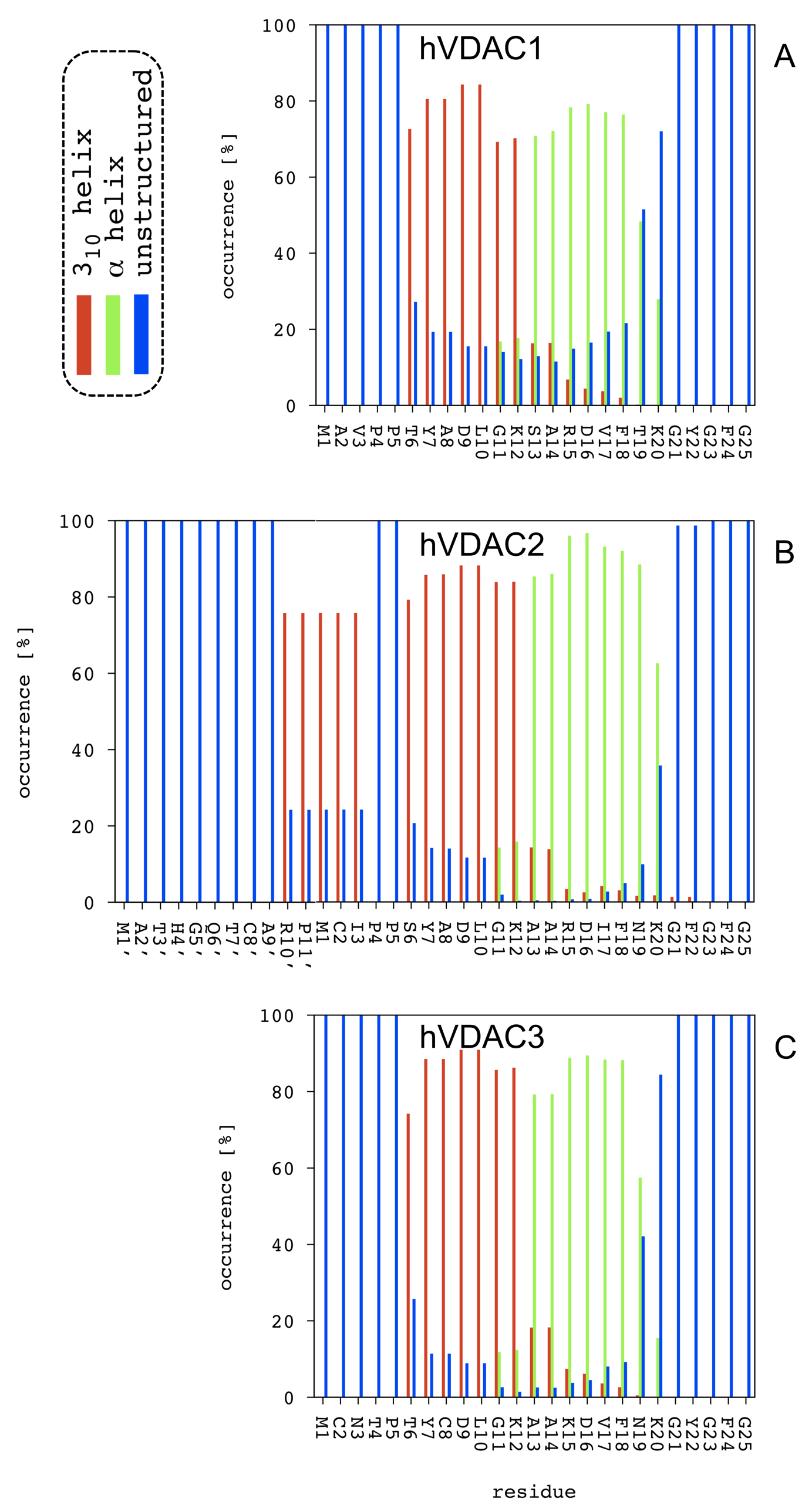

Supplement: Figure S3 — Secondary structure of the N-terminal fragment. Secondary structure is shown for each amino acid residue as the average occurrence of 310-helical, α-helical or unordered conformation over 5 independent MD replicas. The three panels are conveniently placed to reproduce the correct sequence alignment between (A) hVDAC1, (B) hVDAC2 and (C) hVDAC3. (TIFF) [file pone.0103879.s003.tiff]

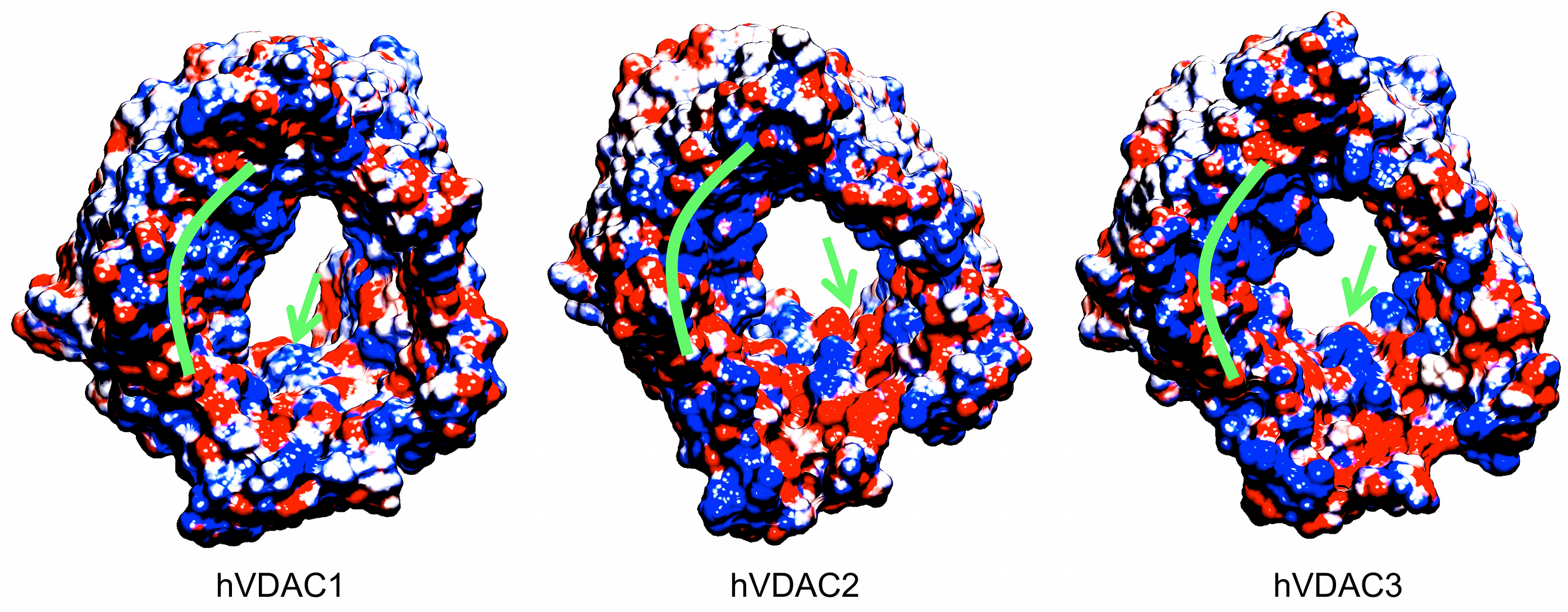

Supplement: Figure S4 — Electrostatic potential surface. The three isoforms are represented from the bottom with positive potential in blue and the negative one in red. The green line represents the position of the N-terminal fragment inside the lumen, whereas the green arrow indicates the area with the most remarkable differences. (TIFF) [file pone.0103879.s004.tiff]
