# Supplementary material for: Charged Residues Distribution Modulates Selectivity of the Open State of Human Isoforms of the Voltage Dependent Anion-Selective Channel
Source: PLoS One. 2014 Aug 1;9(8):e103879. doi: 10.1371/journal.pone.0103879 (PMC4146382; doi:10.1371/journal.pone.0103879)
Supplement: Table S1 — Pearson cross-correlation coefficients for different axes describing the elliptic shape of the channel. (DOCX) [file pone.0103879.s005.docx]

| **Table S1.** Pearson cross-correlation coefficients for different axes describing the elliptic shape of the channel. | | | | | | |
| --- | --- | --- | --- | --- | --- | --- |
| **hVDAC1** | | | | | | |
|  | ***Lt*** | ***Lm*** | ***Lb*** | ***Tt*** | ***Tm*** | ***Tb*** |
| ***Lt*** | +1.00 ± 0.00 | +0.86 ± 0.06 | +0.47 ± 0.26 | -0.64 ± 0.18 | -0.66 ± 0.20 | -0.31 ± 0.31 |
| ***Lm*** | +0.86 ± 0.06 | +1.00 ± 0.00 | +0.70 ± 0.09 | -0.66 ± 0.13 | -0.73 ± 0.13 | -0.43 ± 0.20 |
| ***Lb*** | +0.47 ± 0.26 | +0.70 ± 0.09 | +1.00 ± 0.00 | -0.48 ± 0.13 | -0.63 ± 0.13 | -0.41 ± 0.23 |
| ***Tt*** | -0.64 ± 0.18 | -0.66 ± 0.20 | -0.48 ± 0.13 | +1.00 ± 0.00 | +0.71 ± 0.04 | +0.27 ± 0.20 |
| ***Tm*** | -0.66 ± 0.20 | -0.73 ± 0.13 | -0.63 ± 0.13 | +0.71 ± 0.04 | +1.00 ± 0.00 | +0.67 ± 0.12 |
| ***Tb*** | -0.31 ± 0.31 | -0.43 ± 0.20 | -0.41 ± 0.23 | +0.27 ± 0.20 | +0.67 ± 0.12 | +1.00 ± 0.00 |
| **hVDAC2** | | | | | | |
|  | ***Lt*** | ***Lm*** | ***Lb*** | ***Tt*** | ***Tm*** | ***Tb*** |
| ***Lt*** | +1.00 ± 0.00 | +0.79 ± 0.10 | +0.38 ± 0.22 | -0.16 ± 0.33 | -0.13 ± 0.34 | -0.14 ± 0.47 |
| ***Lm*** | +0.79 ± 0.10 | +1.00 ± 0.00 | +0.59 ± 0.16 | -0.15 ± 0.29 | -0.20 ± 0.28 | +0.26 ± 0.34 |
| ***Lb*** | +0.38 ± 0.22 | +0.59 ± 0.16 | +1.00 ± 0.00 | +0.08 ± 0.24 | -0.01 ± 0.28 | -0.16 ± 0.29 |
| ***Tt*** | -0.16 ± 0.33 | -0.15 ± 0.29 | +0.08 ± 0.24 | +1.00 ± 0.00 | +0.71 ± 0.20 | +0.46 ± 0.11 |
| ***Tm*** | -0.13 ± 0.34 | -0.20 ± 0.28 | -0.01 ± 0.28 | +0.71 ± 0.20 | +1.00 ± 0.00 | +0.68 ± 0.17 |
| ***Tb*** | -0.14 ± 0.47 | +0.26 ± 0.34 | -0.16 ± 0.29 | +0.46 ± 0.11 | +0.68 ± 0.17 | +1.00 ± 0.00 |
| **hVDAC3** | | | | | | |
|  | ***Lt*** | ***Lm*** | ***Lb*** | ***Tt*** | ***Tm*** | ***Tb*** |
| ***Lt*** | +1.00 ± 0.00 | +0.85 ± 0.03 | +0.69 ± 0.09 | -0.71 ± 0.04 | -0.47 ± 0.21 | -0.34 ± 0.23 |
| ***Lm*** | +0.85 ± 0.03 | +1.00 ± 0.00 | +0.85 ± 0.03 | -0.74 ± 0.06 | -0.64 ± 0.14 | -0.55 ± 0.11 |
| ***Lb*** | +0.69 ± 0.09 | +0.85 ± 0.03 | +1.00 ± 0.00 | -0.62 ± 0.09 | -0.59 ± 0.09 | -0.57 ± 0.11 |
| ***Tt*** | -0.71 ± 0.04 | -0.74 ± 0.06 | -0.62 ± 0.09 | +1.00 ± 0.00 | +0.67 ± 0.10 | +0.45 ± 0.16 |
| ***Tm*** | -0.47 ± 0.21 | -0.64 ± 0.14 | -0.59 ± 0.09 | +0.67 ± 0.10 | +1.00 ± 0.00 | + 0.73 ± 0.23 |
| ***Tb*** | -0.34 ± 0.23 | -0.55 ± 0.11 | -0.57 ± 0.11 | +0.45 ± 0.16 | + 0.73 ± 0.23 | +1.00 ± 0.00 |
| Error is given as the standard deviation of 5 independent MD replicas.  Six different distances were chosen to estimate the length of the elliptic axes, namely, longitudinal and transversal to the N-terminal fragment, at three different heights with respect to z-axis of the pore (L: *longitudinal*; T: *transversal*; t: *top*; m: *middle*; b: *bottom*). | | | | | | |
